# Hybridizing mechanistic mathematical modeling with deep learning methods to predict individual cancer patient survival after immune checkpoint inhibitor therapy

**DOI:** 10.21203/rs.3.rs-4151883/v1

**Published:** 2024-03-29

**Authors:** Joseph D Butner, Prashant Dogra, Caroline Chung, Eugene J Koay, James W Welsh, David S Hong, Vittorio Cristini, Zhihui Wang

**Affiliations:** 1 Department of Radiation Oncology, The University of Texas MD Anderson Cancer Center, Houston, TX 77030, USA;; 2 Institute for Data Science in Oncology, The University of Texas MD Anderson Cancer Center, Houston, TX 77030, USA;; 3 Master in Clinical Translation Management Program, The Cameron School of Business, University of St. Thomas, Houston, TX 77006, USA;; 4 Mathematics in Medicine Program, Houston Methodist Research Institute, Houston, TX 77030, USA;; 5 Department of Physiology and Biophysics, Weill Cornell Medicine, New York, NY 10065, USA;; 6 Department of Investigational Cancer Therapeutics, University of Texas MD Anderson Cancer Center, Houston, Texas 77230, USA;; 7 Neal Cancer Center, Houston Methodist Research Institute, Houston, TX 77030, USA;; 8 Physiology, Biophysics, and Systems Biology Program, Graduate School of Medical Sciences, Weill Cornell Medicine, New York, NY 10065, USA;; 9 Department of Imaging Physics, University of Texas MD Anderson Cancer Center, Houston, TX 77230, USA;; 10 Department of Medical Education, Texas A&M University School of Medicine, Bryan, TX 77807, USA.

**Keywords:** Cancer treatment, machine learning, mathematical biomarkers, mathematical modeling, precision medicine, tumor-immune interaction

## Abstract

We present a study where predictive mechanistic modeling is used in combination with deep learning methods to predict individual patient survival probabilities under immune checkpoint inhibitor (ICI) therapy. This hybrid approach enables prediction based on both measures that are calculable from mechanistic models (but may not be directly measurable in the clinic) and easily measurable quantities or characteristics (that are not always readily incorporated into predictive mechanistic models). The mechanistic model we have applied here can predict tumor response from CT or MRI imaging based on key mechanisms underlying checkpoint inhibitor therapy, and in the present work, its parameters were combined with readily-available clinical measures from 93 patients into a hybrid training set for a deep learning time-to-event predictive model. Analysis revealed that training an artificial neural network with both mechanistic modeling-derived and clinical measures achieved higher per-patient predictive accuracy based on event-time concordance, Brier score, and negative binomial log-likelihood-based criteria than when only mechanistic model-derived values or only clinical data were used. Feature importance analysis revealed that both clinical and model-derived parameters play prominent roles in neural network decision making, and in increasing prediction accuracy, further supporting the advantage of our hybrid approach. We anticipate that many existing mechanistic models may be hybridized with deep learning methods in a similar manner to improve predictive accuracy through addition of additional data that may not be readily implemented in mechanistic descriptions.

## Introduction

Immunotherapy has gained significant traction as an effective anti-cancer therapy. By activating and recruiting the patient’s own immune response against tumors, it is often able to achieve greater efficacy with fewer side effects than chemotherapeutic approaches [1]. Commonly, this is accomplished by using a family of monoclonal antibodies known as immune checkpoint inhibitors (ICIs), which function by binding the immune checkpoints anti-programmed death ligand 1 (PD-1), its ligand (PD-L1), or cytotoxic T-lymphocyte-associated protein 4 (CTLA-4) [2], blocking the immune inhibitory effects of these pathways [3]. ICIs have proven to be remarkably successful, and clinical trials on ICIs now account for the majority of all oncological clinical trials [4]. However, it is estimated that only around 40% of cancer patients benefit from ICI treatment [5]. ICI-induced immune activation can also cause significant inflammatory side-effects due to off-target immune interactions [1, 6], which, although uncommon, can lead to patient fatality [7]. Current ICI usage rates are approaching the estimated total population that may receive therapeutic benefit (one meta-analysis reported 39.2% in 2019) [8], but it is unlikely that all treated patients fall within the population that will receive benefit. There remains a significant unmet need for methods to identify patients likely to receive benefit from ICI on a per-patient basis.

To date, research on ICI outcome assessment has been primarily focused on retrospective assessment of response after treating with ICI for a period; these include immune RECIST (iRECIST) [9] and immune-related response criteria (irRC) [10]. These criteria are immunotherapy analogs of Response Evaluation Criteria in Solid Tumor (RECIST) v1.1, which is commonly used for response assessment to more traditional therapies in clinical trial settings. Genomic-based rubrics [11] (e.g., tumor mutational burden [12]), machine learning-based approaches [13], and transcriptomic rubrics [14] have also been studied as potential indicators of therapeutic response, among others [15]. These response evaluation methods, while useful, have traditionally defined response as a change in size of one or more tumors, which may not always be indicative of the true gold standard of cancer treatment in clinical practice: extending patient overall survival (OS) [16–19]. Furthermore, measures based on traditional biomarkers have proven to be inconsistent among varying tumor types and underlying tumor microenvironment conditions [20–23]. We [24–26], and more recently other groups as well [27], have developed straightforward, calculable scores that are indicative of response by the first tumor restaging. Although still retrospective (i.e., applicable only after initiating therapy and observing initial results), these are commonly usable at times earlier after start of treatment than RECIST-like scores, which must wait until a defined volume change threshold is observed.

Our recently published mechanistic mathematical model is grounded in biological and physical phenomena for accurately predicting solid tumor response [24–26]. It is also demonstrated to reliably sort patients into prognostically favorable vs. unfavorable survival groups using a model-derived parameter representing the tumor growth rate at time of first restaging ($\alpha_{1}$; see Methods). Discretion between survival groups (favorable vs. unfavorable) in these studies was done using binary classifier thresholds identified by receiver operator curve (ROC) optimization studies. However, ROC optimization (e.g., C-index, sensitivity, specificity) is more appropriate for discriminating between already-existing conditions [28] than it is for predicting future events, which are subject to stochasticity and thus are more appropriately described as probabilities (e.g., partial likelihoods via logistic regression or COX proportional hazards, or by probability mass functions) than binary sets [29, 30]. However, such probabilistic functions are not readily generable as outcomes from pure mechanistic models.

Recent advances in deep learning (DL) methods have identified powerful approaches to generate DL-predicted partial likelihood curves using properly trained artificial neural networks. Here, we present a new approach where we have hybridized our existing mechanistic model with a DL partial likelihood-based prediction method. This enables individual patient survival prediction based on a combination of mechanistic model-derived parameters combined with additional standard-of-care clinical markers and clinical information that may be relevant to treatment outcome but not be easily included in mechanistic (that is, equation-based) models (e.g., age, sex, race, disease stage) to achieve higher per-patient predictive accuracy that when using only the mechanistic model or only DL models trained on clinical measures. This is a powerful new approach to feature construction [31, 32], where some DL input features are constructed using advanced mechanistic modeling (note that it could be argued that some clinical features are also constructed, e.g., age = date of on-study minus date of birth). Instead of only testing discrimination between survival groups (e.g., binary classifier-based sorting), this approach achieves more global data fits (that is, they give quantified predicted probabilities over the full time range studied) that simultaneously optimize discrimination and calibration (not only sorting into binary survival groups, but also allowing quantification of how close the predicted event likelihood is to the observed event), based on statistics such as the Brier score [33, 34]. As a result, it enables probability-based, time-dependent survival prediction (or more generally, time-to-event prediction) on an individual basis. Moreover, this approach provides a marked improvement over our prior efforts to predict patient survival using population-based statistics-based parameters, which may be accurate when applied across a large population but not necessarily for an individual [33].

## Methods

The results presented herein were obtained using a two-step modeling process, where we first applied an established mechanistic model of ICI therapy in solid tumors to a retrospective patient cohort and then combined the output from the mechanistic model with additional, often non-mechanistic patient data to train an artificial neural network (ANN) for prediction of patient survival probabilities. This approach generates functions that describe the probability of a patient death (an event) over time. In a more general sense, this is time-to-event prediction, where we define start time is the time of first administration of ICI drug (*t*_0_), event is defined as patient death, and patients are censored after time of last follow-up. We are aware that multiple methods have been demonstrated to make time to event predictions, including continuous methods such as Cox proportional hazards [35] or DeepSurv [36], and also based on discrete methods including logistic hazards [35, 37], probability mass function (PMF) [35], and PMF w/competing risk (DeepHit) [38]. In this work, we will focus on the logistic hazards method because this approach avoids constant hazard ratio assumptions (e.g., COX) while offering superior performance than other discrete methods with small sample sizes [35], and our purpose here is to test the hypothesis that mechanistic + clinical parameters will result in superior DL model performance, as opposed to comparing performance across methods.

## Mechanistic mathematical modeling

**Figure 1:**
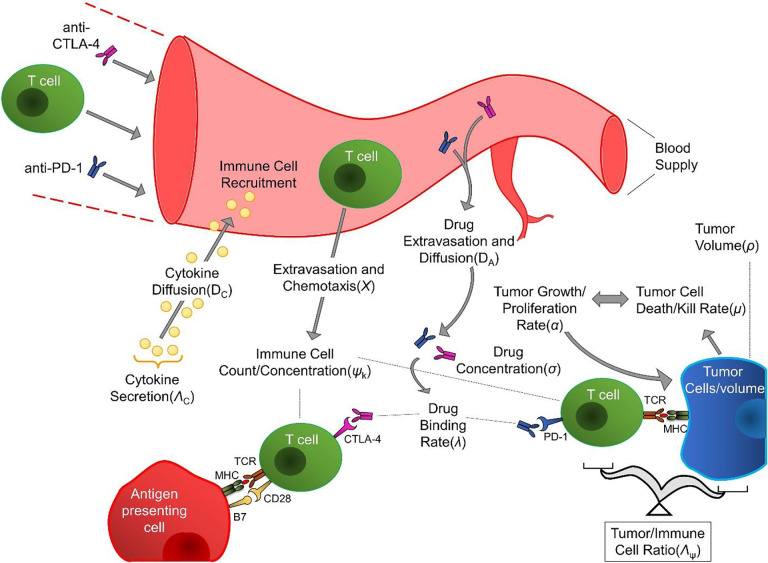
Key biological and physical factors and processes included in the mechanistic mathematical model.

Mathematical descriptions of the mechanistic biological and physical processes underlying checkpoint inhibitor therapy may be informed through clinically measurable quantities to predict treatment response and patient outcome. Moreover, these biological and physical phenomena are mechanistically linked through physical laws and key feedback mechanisms. Based on this understanding, we have developed a mathematical model [24] that describes the total tumor burden (ρ) over time, and is built upon 1) the key, mechanistic biological factors or processes (e.g., concentration of therapeutic T cells $\left(\psi_{k}\right)$, intratumoral concentration of immunotherapy antibody (σ), cytokine secretion $\left(\Lambda_{C}\right)$, and ratio of immune to tumor cells over time $\left(\Lambda_{\psi }\right)$ and 2) the physical factors or processes (e.g., rates of untreated tumor cell proliferation $\left(\alpha_{0}\right)$ and death due to ICI therapy (μ), binding of antibody to targets (λ), specific death rate of cancer cells $\left(\lambda_{p}\right)$, chemotaxis (χ), diffusion of antibodies and cytokines $\left(D_{A},D_{C}\right)$, and mass conservation that underlie ICI immunotherapy intervention (Fig. 1). By mathematically linking the relationships between these processes, our model quantifies their combined effects (and the feedback processes between them) on the time-dependent change in tumor burden (ρ) under immunotherapy intervention.

The model derivation process has been extensively detailed previously [24–26]; we only present a brief overview of the key mechanisms shown in Fig. 1 and their mathematical descriptions. Fick’s law descriptions are used to obtain steady-state diffusion of antibodies (A) and cytokines (C) within the tumor and the balances of antigen-antibody interaction and cancer cell-cytokine concentrations within the tumor microenvironment (TME) according to

(1) $$D_{A}\cdot \nabla^{2}\sigma =\lambda \cdot \sigma \cdot \rho$$

and

(2) $$D_{C}\cdot \nabla^{2}C=-\Lambda_{C}\cdot \lambda \cdot \sigma \cdot \rho ,$$

respectively. The concentration of viable tumor cells over time is a function of the tumor’s intrinsic growth rate reduced by the tumor cell kill rate due to antibody binding (i.e., checkpoint inhibitors binding to their respective ligands) and the time history of antibody uptake and binding within the tumor, and may be written as

(3) $$\frac{\partial \rho }{\partial t}=\alpha_{0}\cdot \rho -\lambda_{p}\cdot \rho \cdot \psi_{k}\cdot \int_{0}^{t}\lambda \cdot \sigma \cdot \rho \cdot dt^{\prime }.$$

The time-dependent intratumoral concentration of therapeutic immune cells is a function of chemotaxis-mediated migration into and within the tumor, as a result of cytokine signaling and immune cell coupling with tumor cells as follows:

(4) $$\frac{\partial \psi_{k}}{\partial t}=-\chi \cdot \nabla \cdot \left(\psi_{k}\cdot \nabla \cdot C\right)+\Lambda_{\psi }\cdot \frac{\partial \rho }{\partial t}.$$

Applying reasonable assumptions and solving these 4 equations leads to our master equation that mechanistically describes tumor burden over time as an outcome of immunotherapy intervention:

(5) $$\frac{d\rho^{\prime }}{dt}=\rho^{\prime }\left(\alpha_{0}-\mu +\Lambda \mu \right)+\rho^{\prime 2}(-\Lambda \mu ),$$

Where $\rho^{\prime }$ is tumor volume normalized by the tumor volume at t=0 (i.e., $\rho^{\prime }=\rho_{t=n}/\rho_{t=0}),\;\alpha_{0}$ is the intrinsic (baseline) tumor growth rate without treatment intervention, and the key model parameters (these are *mathematical biomarkers* (MBs)) are the tumor kill rate (μ) by immunotherapy and patient anti-tumor immune state (Λ), which we defined as the coupling of immune cell activity and the tumor cell kill (i.e., immunogenicity of a tumor) scaled by the ratio of tumor cells to intratumoral immune cells at the time of treatment. For simplicity moving forward, we will drop the prime on $\rho^{\prime }$, so that equation 5 becomes

(6) $$\frac{d\rho }{dt}=\rho \left(\alpha_{0}-\mu +\Lambda \mu \right)+\rho^{2}(-\Lambda \mu ).$$

For our initial mechanistic model (equation 6), patient-specific estimation of the total patient tumor burden, mathematical biomarkers μ and Λ, and associated intrinsic growth rate $\alpha_{0}$ (that is, the average pre-treatment growth rate across all measured tumors) was performed for each patient from a previously-obtained patient cohort (n=93; patients treated with ipilimumab on clinical trial NCT02239900) from CT imaging measurements after treatment initiation. Detailed patient characteristics tables may be found in [24] (Tables 2 for the institutional validation cohort and S2 therein). $\alpha_{1}$ (i.e., growth rate between start of treatment and the time of first (1^st^) restaging $\left(t_{1}\right)$) was also calculated by fitting the short-term model solution $\left(\rho (t)\approx e^{\alpha_{1}\cdot t}\right)$ between the CT imaging measured tumor burden (ρ) measured at time of treatment initiation (t=0) and at the time of first restaging $\left(t_{1}\right)$, calculated as $\alpha_{1}\approx ln\left(\rho \left(t_{1}\right)\right)/t_{1}$.

**Figure 2:**
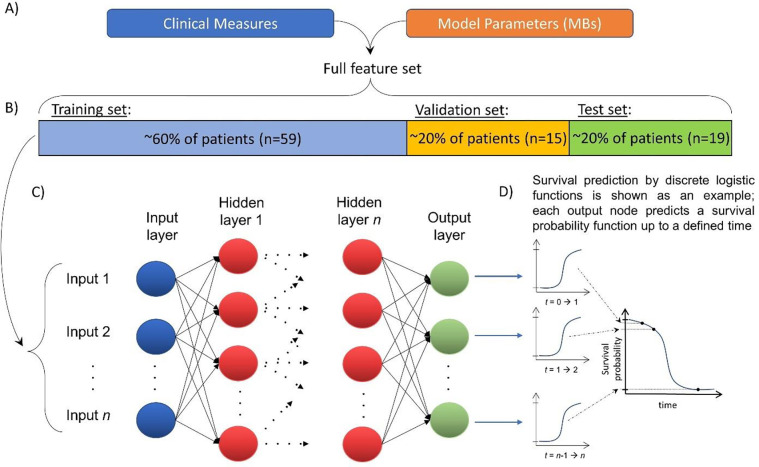
Overview of hybrid modeling approach. A) Clinical and mathematical biomarkers (MBs) are combined into B) a full feature set for training C) an artificial neural network to predict patient survival probabilities (note that all ANNs used in this study had contained a single hidden layer). Roughly 20% of the patients are withheld from the training process as a test set for evaluating predictions after the DL model training is complete. The ANN outputs a series of logistic functions that predict the likelihood of a death event being observed for each discrete time increment; D) these are then combined into full survival probability curves (probability of survival is 1 minus probability of death) as shown in Fig. 3.

After solving for these key parameters from the mechanistic mathematical model, we combined them with a set of other clinical measures (Table 1) to train and validate a deep learning (DL) model for predicting individual patient survival. The overall approach is shown in Fig 2. At this time, we have only included data that were readily available and complete (that is, no missing measurements for any patient) in order to test our hypothesis that a hybrid (mechanistic + DL) approach using both model-derived and clinical data may improve accuracy over either method alone; however, we recognize that it is unlikely that this constitutes the set of model + clinical measures that would enable the DL model to achieve the maximum theoretical predictive accuracy if all possible measures were available. By using a patient cohort collected as part of an in-house clinical trial, we have been able to collect a broad set of individual patient parameters that we hope captures much of the pertinent information about patient prognosis; however, this decision has made it difficult to find matching external validation sets, and as a result we have generated a validation set by withholding a subset of the patients from model training. Note that the neutrophil to lymphocyte ratio (NLR; a commonly reported clinical measure) was not included in our DL model to avoid collinearity with neutrophil and lymphocyte counts and improve the accuracy of the feature importance analysis.

## Deep learning modeling

### Modeling approach overview

Survival predictions generated herein are based on a methodology first published by Brown [39], who observed that the standard Kaplan-Meier survival curve may be thought of as a set of discrete binary states (where each patient has a unique probability of being alive or deceased at each ‘step’ in the curve). Thus, a Kaplan-Meier curve may be approximated by a series of binary functions over time. Commonly, binary classification prediction is done using logistic regression functions. By subdividing the time domain into a series of discrete windows, each containing a set of observed patient deaths (these are the events to be predicted), a unique logistic function may be fit to each concordant time increment by minimizing its loss function (Bernoulli’s negative log likelihood in the case of logistic regression with right-censored data) to the data subset contained within that increment. Machine learning (ML) is a natural choice for this approach, as binary classification is usually based on logistic regression when done with ML/DL [35], and ML/DL platforms are robust tools to process many types of data.

### Data preprocessing

**Figure 3:**
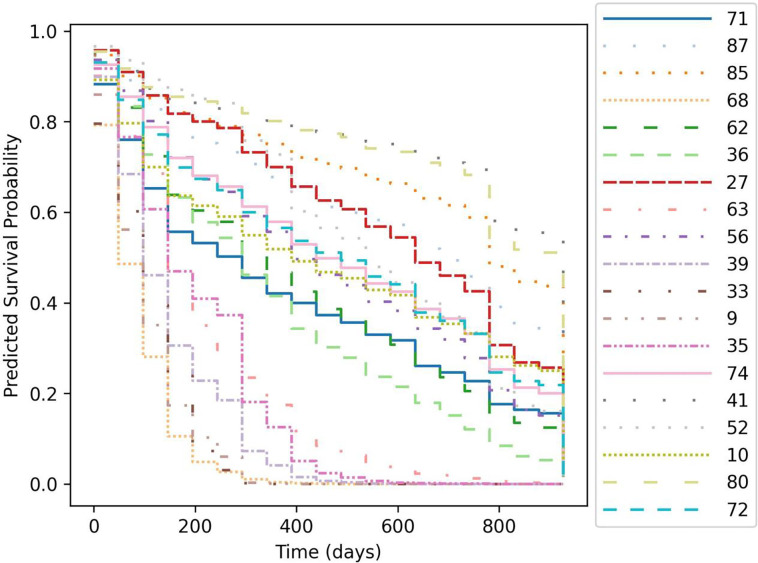
Per-patient predicted survival curves for the test set (withheld from model training) of n = 19 randomly selected and ordered patients; curve numbers correspond to the de-identified patient number (1 – 93) assigned to each patient. The event-time concordance was found to be C-index = 0.789 across the full test set, with IPCW Brier score = 0.123. Predicted survival curves from are shown split into 4 separate plots for increased readability in supplemental **Fig. S1**.

Data were pre-processed using standard ML approaches; briefly, categorical data were one-hot encoded, data were split into training, validation, and test sets as shown in Fig. 2B, and then continuous data were normalized using the StandardScalar function from scikit [40] based on the training set only. It can be observed in Table 1 that the distribution of race is highly unbalanced; however, no feature balancing (e.g., resampling via oversampling, SMOTE, SMOTE-N, etc. [41] or cost-sensitive weighting [42]) was performed in this study; implications of this approach are further examined in Discussion. To validate the model, k-fold validation was implemented, where continuous data were renormalized for each fold (normalization should be done based on only the unique training set chosen for each fold to avoid ‘data leakage’ from the validation and test sets into the training set). Finally, data was subdivided into time increments (n = 20 increments was used in this study) in order to generate a unique DL-generated logistic curve for each increment (Fig. 2D); each ‘step’ in the curves shown in Fig. 3D corresponds to one discrete time increment [35]. Time discretization also enables more accurate prediction within each time window by providing a method to account for censoring events while also reducing the potential skewing effects of concentrated events (in our case, death or censor) observed at distant times (before or after the time increment) [35]. We used an equidistant time discretization scheme [35] for the results presented here (alternatively, this can also be done based on event distribution via Kaplan-Meier quartiles). All data preprocessing was conducted using scikit [40], pandas [43], NumPy [44], and torchtuples, and plots were generated using matplotlib [45] and Plotly [46].

### Model training and statistical analysis

After data handling, a multilayer perceptron ANN was trained using the logistic hazards time-to-event modeling [35, 37] functionality from the PyCox package [35, 47] and PyTorch. For the results presented here, the ANN was constructed with a single hidden layer, rectified linear unit (ReLU) activation functions were optimized by Adaptive Moment Estimation (ADAM), and training epochs were terminated when the error function of the validation set was minimized. For simplicity, the number of nodes in the hidden layer was selected to be the arithmetic mean between nodes in the input layer (number of features) and the output layer (n=20), rounded down to the nearest integer if needed. Hyperparameters were tuned using randomized search with cross validation using scikit-learn 1.4.1 modules BaseEstimator and RandomizedSearchCV with a target of minimizing the IPCW Brier score (see below), followed by manual hyperparameter tuning as needed. Other hyperparameters used for the hybrid ML + mechanistic model include batch normalization, a dropout rate of 0.2 (prevents overfitting), per-epoch batch size of 50 (roughly 85% of the training set), a learning rate 0.07, and early stopping was enabled (for this small study, all training cycles finished under 512 epochs). No feature selection or elimination was performed in this study; all available features were included in all analyses regardless of importance. No tuning was performed for the randomized data grouping (patients being assigned to train, validation, or test sets) shown in Fig. 2B.

At all times, the ANN training algorithm remained agnostic to the test set, which was then used to evaluate the predictive accuracy of the trained neural network after training was complete. The trained ANN was tasked with predicting survival on each patient in the test set; individual patient parameters for the test set are shown in **Table S1**. The accuracy of per-patient survival predictions was then assessed using 1) an event-time concordance index [48] (similar to the standard C-index, but calculated over time based on an extension of the C-index with right censoring proposed by Harrel [49]), 2) the time-dependent error in predicted hazard functions using the inverse probability of censoring weighting (IPCW; a method of accounting for right-censored events in the data by approximating the score based on the inverse probability of censoring) Brier score (a method of scoring accuracy based on comparing predicted likelihoods of an event vs. if it was observed or not that also considers time between predicted and observed events) [29, 50], and 3) the IPCW negative binominal log-likelihood [50] (NBLL; a log-likelihood weighted by the inverse of the censoring distribution). Both IPCW Brier score and IPCW negative binomial log-likelihood are integrated across the time dimension to provide a single score that is reported herein (see **Fig. S2** for more details). Note that IPCW NBLL is similar to the loss function for DL model training (the mean negative log-likelihood of the hazard parameterization model [35]), but weighted by the inverse probability of censoring.

In order to test the stability and reliability of the trained hybrid mechanistic + DL model and associated feature importances in the ANN, we performed a k-fold validation where the steps shown in Fig. 2B-D were repeated for all permutations of training, validation, and test sets by dividing the data into n = 5 groups of ~20% patients in each group (n = 20 total folds). All hyperparameters were held constant for each fold. Model stability was assessed by comparing the loss function (here, the loss function is the mean negative log likelihood as described in [35]) for the validation cohort (the set that determines when model training stops) for each k-fold; note that for this application, error in the loss function for the held-out data set is preferable to the C-index or other statistical evaluators of model accuracy, which may not be appropriate for distinguishing between models [51]. Stability of the time-dependent C-index was also examined across folds, and descriptive statistics describing variations between folds were calculated.

## Characterizing the trained hybrid deep learning model by feature importances

Finally, we sought to quantify the feature importances; that is, how much each model input (these are *features*: both mechanistic model parameters and clinical measures) contributes to how the artificial neural network makes predictions based on 1) loss function minimization (as a surrogate for direct statistical accuracy, under the assumption that minimizing the loss function maximizes accuracy) and 2) statistical methods that directly calculate rubrics of model accuracy. This is possible because the DL model is trained by minimizing the negative log-likelihood of the hazard parametrization model loss function, while model predictive accuracy may be directly evaluated by using event-time concordance, ICPW Brier score, and ICPW negative binomial log-likelihood. It is important to note that these two approaches provide complimentary but distinct information, as the loss function is minimized based on the training and validation sets (thus evaluating importances based on how the trained neural network makes predictions), while statistical evaluation of prediction accuracy is performed using the test set (these data are withheld during model training, enabling evaluation of feature importance evaluation based on the accuracy of model predictions; see Fig. 2), and feature importance analysis may be performed independently against each of these functions. The result is a two-pronged approach to robustly evaluate if both clinical and mechanistic model-derived measures are needed within a single DL model for maximum predictive accuracy. Because the DL method used here results in a discrete logistic regression curve for each time increment (e.g., Fig. 2D), there is an associated feature importance for each distinct logistic curve (corresponding to each time increment). This is analogous to classification models where a unique output node is generated for each possible label, and likewise features may contribute in different ways to each label. As a result, feature importance analysis yields an array sized [number of features] × [number of outputs]. These may either be examined individually for each output node (in our case, a single logistic function corresponding to one ‘step’ in Fig. 3), which we refer to herein as local feature importance, or they may be combined across all output nodes (we have summed them here, but averages could also be used) to determine their effects on the full model; we will refer to these as global importances.

We used standard methods to study feature importances from the ANN trained via loss function minimization, including several back propagation methods: integrated gradients [52], integrated gradients with smoothing [53], DeepLift [54], and DeepLitfSHAP [54, 55] (approximates SHapley Additive exPlanations (SHAP) values using the DeepLift approach); feature ablation (a method of determining importances via feature perturbation); and SHAP values [55] (an approach derived from game theory that attempts to maximize the gain from each player while ensuring the gain is at least as much as each player would yield independently). In order to directly examine how much each feature contributes to the statistical accuracy when the trained ANN makes survival predictions on new patient data, we passed the event-time concordance evaluator, IPCW Brier score evaluator, and IPCW negative log-likelihood evaluator to a feature permutation algorithm using a wrapper from the eli5 package, which estimates importances using a leave-one-out approach and any black-box scoring rubric. Importantly, in order to preserve the integrity of the trained ANN between calls to different importance calculation algorithms, we first saved the trained ANN to the hard drive immediately after training was complete, and then the original ANN was re-imported in between each importance calculation. Feature importance calculations were done using the Captum [56], SHAP [55], and eli5 [57] packages.

## Results

### Hybrid model predictions and statistical analysis

Parameters μ,Λ and $\alpha_{1}$ from the mechanistic mathematical model (these are mathematical biomarkers: MBs) have been shown to be predictive of patient response (see [24, 26] for details). Model parameter $\alpha_{1}$ was also found to predict patient survival using only non-invasive imaging data acquired as part of standard-of-care up until the first restaging assessment (P=0.0067) by sorting patients into prognostically “favorable” (relatively slower growing lesions; $\alpha_{1}\leq 0.002$ day^−1^) and “unfavorable” (rapid growth; $\alpha_{1}>0.002$ day^−1^) groups [24]. These previously published data were combined with another set of newly collected standard clinical data; these are shown in Table 1. After hyperparameter tuning, survival curves were generated for the test set (n=19 patients) using the trained ANN; these are shown in Fig. 3. Assessment of per-patient prediction accuracy revealed an event-time concordance for the full test set of C-index = 0.789, while the IPCW Brier score [29, 50] was 0.123 and the IPCW negative binominal log-likelihood [50] was 0.397. Moreover, event-time concordance was reduced when the model was trained using only clinical (C-index = 0.731) or only mechanistic model parameters (MBs; C-index = 0.764) after corresponding hyperparameter optimization (see Methods) and with random seeding held constant to ensure identical patient distribution among training, validation, and test sets for each case (only MBs, only clinical, or hybrid MBs + clinical), supporting our hypothesis that predictive accuracy is maximized when both clinical measures and MBs are used together. Similarly, IPCW Brier score and IPCW negative binominal log-likelihood increased to 0.168 and 0.491, respectively, when only clinical measures were used, while they were found to be 0.182 and 0.547 when the DL model was only trained on mechanistic model parameters.

k-fold validation was performed to assess the reliability and stability of the trained neural network for the hybrid (MBs + clinical data) model. Additionally, to evaluate the hyperparameter values selected, we held these values constant across all folds. As discussed in Methods, the appropriate k-fold validation rubric is the loss function used to train the ANN (the mean negative log likelihood [51] as described in [35]). Loss for the validation set using standard trained model (the ANN that all subsequent analysis herein was performed on, which is based on random selection of training, validation, and test sets) was found to be 2.823. Loss for the k-fold validation ranged 1.996 – 9.059 (median = 2.606 and mean = 3.067). It is likely that the 9.059 value represents an outlier where the hyperparameters chosen did not result in a well-trained model, as the second highest loss was found to be 4.661 (if this outliner is removed, median = 2.604 and mean = 2.752). Similar results were observed for the DL model trained on only clinical data or only MBs.

### Feature analysis by evaluating the trained neural network

**Figure 4:**
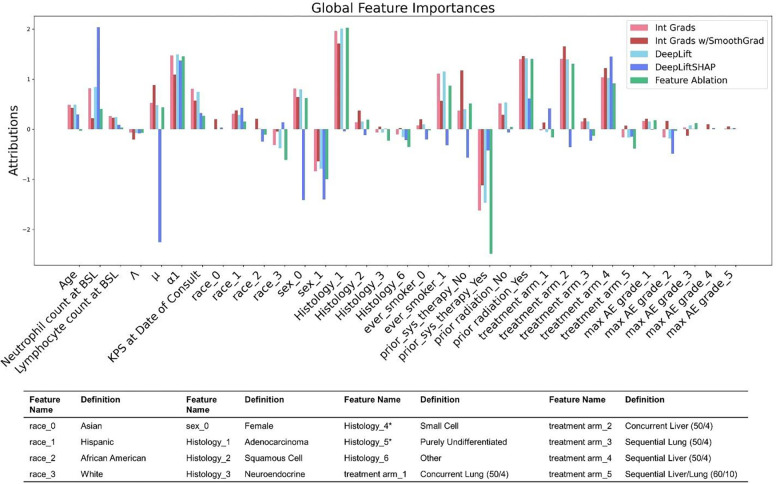
Global feature importance analysis by backpropagation and feature ablation methods. Importances for each feature were calculated using several backpropagation methods (integrated gradients, integrated gradients with smoothing, DeepLift, and DeepLiftSHAP) and also using feature ablation (a leave-one-feature-out method). Bars depict the summed importance across all predicted time increments (see methods and Fig. 2D), and sign indicates a positive or negative association with increased likelihood of seeing an event (in our case, patient death). BSL = baseline, AE = adverse event. *After screening for patient eligibility no Small Cell or Purely undifferentiated histology patients were included in the modeling study.

In order to assess how much each input measure (mathematical model parameters or clinical measures) influences how the deep learning model makes predictions, global feature importance analysis was conducted via multiple methods; results from backpropagation and feature ablation methods are shown in Fig. 4. Each bar represents the sum of importances across all time increments for the associated analysis. Parameters with highest global feature importances include the mechanistic model-derived MBs tumor kill rate (μ) and growth rate at first restaging $\left(\alpha_{1}\right)$, and clinical parameters neutrophil count at baseline, prior systemic therapy, prior radiation therapy, and ever smoker, as well as some histologies and treatment arms. Maximum adverse event and lymphocyte counts were found to have lower importance. Likewise, the categorical feature race was seen to have minimal importance; this is likely due to the imbalanced race distribution seen in Table 1. This analysis may also be performed for each individual time increment (instead of summing across all time increments as shown here) to examine trends in local feature importances over time; examples of these are shown in **Fig. S3**.

**Figure 5:**
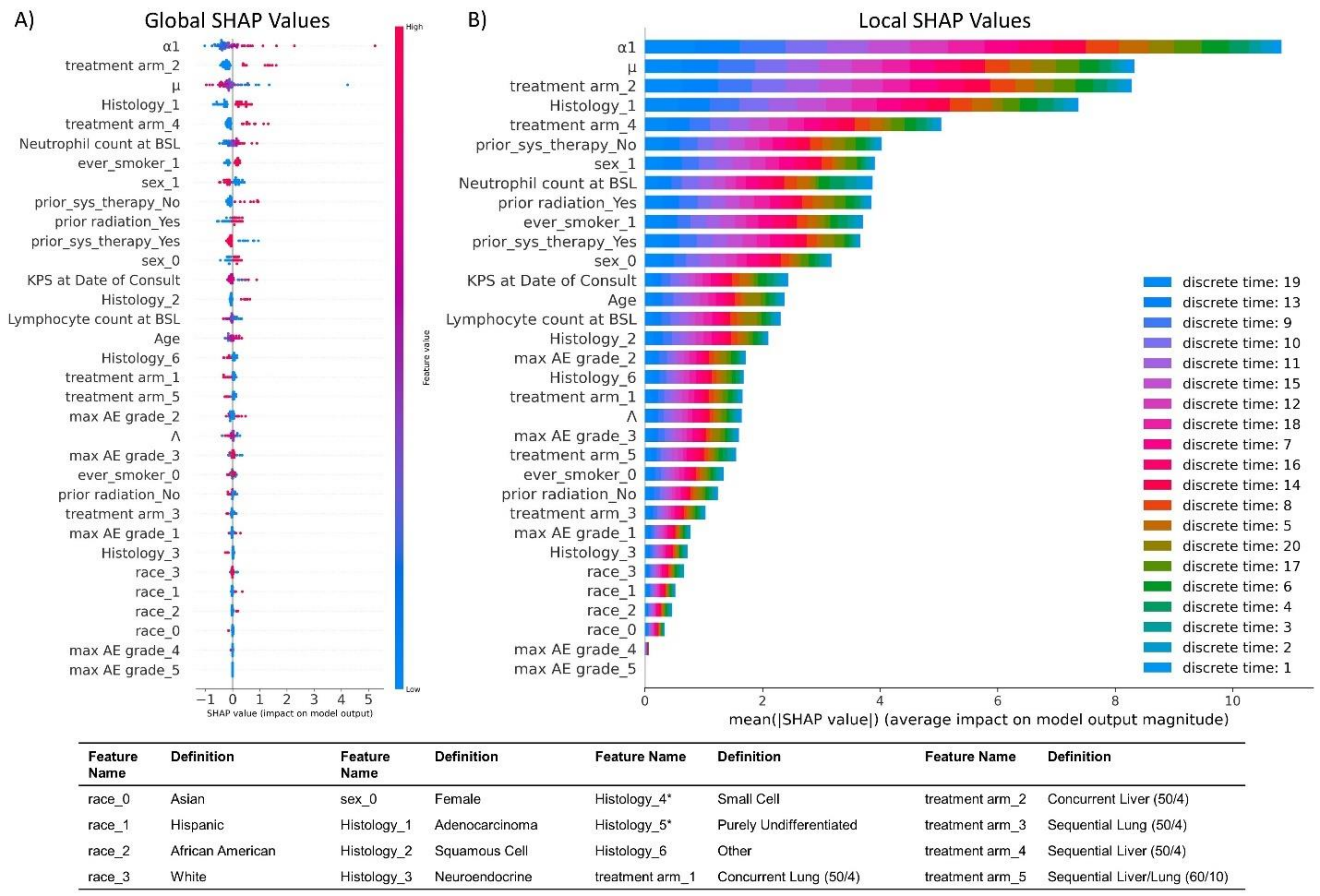
Feature importances by SHAP. A) Global feature analysis reveals feature importance ranking based on the architecture of the trained ANN across all discrete time intervals; each point represents one patient from the training set. Global values were calculated as the average importance across all time steps. B) Local feature analysis gives insights into feature importance weighting for ANN predictions across each time step for the training set; each cut number refers to one time discretization, numbers are in the order of increasing time and corresponds to one step observed in Fig. 3. Feature ordering (y-axis) differences between A and B are because the sign was included when calculating the average in A, while absolute values are summed in B; however it may be observed that ordering trends are similar among both plots. Legend ordering depicts largest to smallest (descending) local importance, and is also shown left to right on each bar in B. BSL = baseline, AE = adverse event. *After screening for patient eligibility no Small Cell or Purely undifferentiated histology patients were included in the modeling study.

Global feature importance analysis via SHAP routine DeepExplainer revealed similar results (Fig. 5A), with tumor kill rate (μ) and growth rate at first restaging $\left(\alpha_{1}\right)$ derived from the mechanistic model ranked highly. SHAP also indicated similar importance rankings of the clinical measures (Fig. 5) identified to have highest importance via backpropagation methods (Fig. 4). Local feature importance analysis was also performed (Fig. 5B), and time steps were ranked by magnitude of contribution to global importance values at each time increment (corresponding to one step in the curves in Fig. 3); local importance contributions to global importances are listed in descending order in Fig. 5B legend. A general trend may be observed where largest local importances are observed towards median times, while lower importances are observed towards early or late times.

### Feature analysis based on statistical accuracy of predictions made with the trained ANN

The previous importance analyses were focused on how features are used by the ANN to make predictions by considering loss function minimization (on the training and validation sets) to be a surrogate for predictive accuracy. While this is reasonable for training the model (wherein the validation set was used to test ANN predictions during training), feature importances determined by evaluating the architecture of the ANN (e.g., backpropagation) only consider the weighting of features across all nodes, without directly assessing any new predictions made by the model on data that it is agnostic to until after training is complete. Prediction accuracy is the primary goal of such modeling efforts, so we will also examine feature importances based on the statistical accuracy of predictions made by inputting a new data set into the trained ANN (that is, without directly examining the ANN). This can be done using the previously-discussed estimators of overall per-patient prediction accuracy, the event-time concordance and Brier score-based methods, via permutation analysis, and is readily accomplished using the eli5 package (see Methods). Of these, we have focused on IPCW Brier score, which considers both the order of event predictions (their concordance) and if events are observed within the same window they are predicted, thereby penalizing correctly ordered predictions that are farther away from the observed timing; note that Brier score is a measure of error, so accuracy is maximized when this score is minimized.

**Figure 6:**
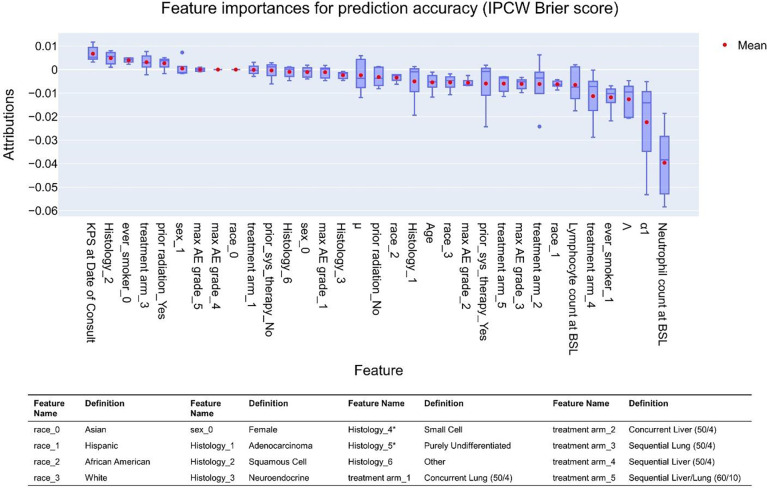
Feature importances based on statical accuracy of predictions made on a withheld data set (test set) by IPCW Brier score. Features are sorted based on the mean of importance attribution, ascending left to right (increasing performance occurs when Brier score is minimized). Boxes represent upper and lower linear quartiles, the dividing line shows the median (middle quartile), whiskers show range excluding outliers, and the mean is shown by a red point. BSL = baseline, AE = adverse event. *After screening for patient eligibility no Small Cell or Purely undifferentiated histology patients were included in the modeling study. Plots for event-time contradance and PICW NBLL are shown in **Fig. S4**.

This analysis was performed on the test set (as opposed to previous importance analysis based on the training set; see Fig. 2), as true predictions can only be made using the test set (that is, the model is only agnostic to this data set after training). As shown in Fig. 6, the features contributing the most to increasing the accuracy of model predictions are a combination of clinical measures (e.g., neutrophil count at baseline, ever smoker, lymphocyte count) and parameters derived from the mechanistic mathematical model ($\alpha_{1}$ and Λ). Notably, the mechanistic model-derived parameter Λ (anti-tumor immune state) is ranked higher in importance based on statistical accuracy than when based on its weighting in predictions made with the ANN in the previous section. Similar feature rankings for predictive accuracy were observed when evaluated based on event-time concordance and IPCW negative binomial log-likelihood; these are shown in **Fig. S4**. Note that, because our rubrics for evaluating predictive accuracy are taken across all time increments, we only examine global importances in this case.

## Discussion

Event-time concordance was found to be C-index = 0.789 in the test set, which we take to be a satisfactory value for a model trained on such a small dataset. This concordance is improved over event time concordance when using only clinical (C-index = 0.731) or only mechanistic model parameters (MBs; C-index = 0.764) alone, and comparable rankings were observed based on Brier scoring. We take this as good evidence that our approach of hybridizing mechanistic model-derived parameters with clinical measures represents a reliable method to improve per-patient survival prediction based on deep-learning methods.

It can be observed in Fig. 3A that some predicted survival curves cross over one another (i.e., there is a lack of ‘one-to-one’ correspondence between the ordering of certain underlying logistic hazard curves (Fig. 2D) over time), which has the effect of lowering the C-index due to only a portion of these curves be in correct concordance. This time-dependent discordance in our predicted survival curves is a natural result of our choice of prediction based on discrete functions (in this case, logistic hazard curves); one may instead choose to use continuous modeling approaches (e.g., based on proportional hazards models, such as Cox [35] or DeepSurv [36]) that avoid this behavior [48]. However, it is likely that covariates and risk may vary among patients as a function of time, and avoiding the restriction of complete concordance common to relative hazards methods is likely advantageous in many cases.

Concordance-based evaluators, which score based on discrimination (prediction distribution vs. measured outcomes), offer the advantage of being more directly relatable to more commonly reported statistics (e.g., area under the ROC curve) and being usable when survival functions are not available, while Brier score-based approaches offer the advantage of evaluation based on both discrimination and calibration (how close the prediction was to the measured outcome). In our study, IPCW Brier score (similar to mean squared error) was found to be 0.123, which is reasonable but leaves room for improvement. The IPCW negative binominal log-likelihood of 0. 0.397 is in reasonable agreement with the IPCW Brier score. We expect that this can be improved by collecting additional clinical measures and data from more patients (see below for further discussion); however, this initial study offers evidence that a hybrid mechanistic modeling + deep learning approach can offer improved per-patient survival prediction accuracy over either approach alone. Plots of IPCW Brier score and negative binomial log-likelihood over time are shown in **Fig. S2A**, where it can be observed that error in prediction accuracy decreases with increasing time. It should also be noted that the extreme tails of these curve may be disregarded, as they correspond to the trivial cases where all patients are predicted to be alive when time *t*=0 or all patients are predicted to be dead as t→∞.

Global feature analysis revealed that the clinical measures neutrophil count at baseline, prior systemic therapy, prior radiation therapy, and ever smoker, some histologies and some treatment arms, and the model-derived MBs tumor kill rate (μ) and growth rate at first restaging $\left(\alpha_{1}\right)$ are weighted highest in the DL model by backpropagation methods (Fig. 4), which is similar to feature ranking by SHAP (Fig. 5A). The consistently high importance assigned to neutrophil count at baseline is to be expected, as high neutrophil counts are reported to associate with poorer survival (and thus more death events) with ICI therapy [58]. The different behavior observed from the GradientShap method (Fig. 4) may be attributed, at least in part, to the fact that this algorithm performs calculations with a reference baseline of the training distribution, while the other backpropagation methods use a reference baseline of zero.

Feature importance rankings based on statistical evaluation of accuracy of model predictions are similar to those observed when examining features based on the architecture of the trained ANN. Both mechanistic model parameters and clinical parameters contribute to the accuracy of model predictions, as supported by both feature analysis and event-time concordance index, which was higher than when only clinical or only mechanistic model-derived features were used. Similarly, IPCW Brier scores and IPCW negative binomial log likelihood scores were lowest in the hybrid clinical + mechanistic model, with increasing error when the model was only trained on mechanistic model derived values or clinical data. Interestingly, baseline neutrophil count was seen to have a greater contribution to the accuracy of predicted patient death events than baseline lymphocyte counts; this implies that if we had alternatively used the commonly reported neutrophil to lymphocyte ratio, the numerator (neutrophils) may have dominated much of the observed prognostic power. It has been reported that baseline neutrophil count may serve well on its own as a prognostic indicator of patient prognosis under ipilimumab treatment [59], and our analysis supports this assertion, at least for the cohort examined in this study.

The race feature category was highly skewed (Table 1), which can cause suboptimal training due to unbalanced training, validation, and test sets (it is likely all race categories may not be represented in all sets), and also because the model may not receive sufficient exposure to underrepresented classes. In classification models, this is commonly corrected by adding additional cases of the underrepresented classes, for example through oversampling (adding copies of the underrepresented patients, which balances the data but does not impart additional information to the DL training algorithm) or methods based on the Synthetic Minority Oversampling Technique [41] (SMOTE; a method of generating new data by interpolating within the feature space of the minority classes), or through undersampling (removing some of the overrepresented class) [60, 61]. Often, oversampling of the minority set is combined with undersampling the minority set [41]. Cost-sensitive methods have also been proposed. Commonly, instances are weighted by the square root of the reciprocal of instances in the training set [42, 62, 63], and methods based on dynamic weighting adjustment [64–66] or loss functions that reflect class balance [66–69] have also been proposed. However, in practice this is usually reserved for classification models (and is principally used in cases where the outcome of interest is unbalanced, as opposed to the input features) and has been reported to be detrimental for logistic regression approaches [70].

Alternatively, rare categorical features are commonly grouped via feature engineering approaches such as binning, where instances occurring below a threshold are “binned” into a single category (in our case, this might be white vs. non-white) or through feature-reducing embedding methods (there is a commonly referenced guideline of selecting the number of embeddings based on the 4^th^ root of the number of categories based on [71], but this not practical in our case), but we have elected to forgo this as well due to the small number of race categories (n = 4). Ultimately, we elected to simply use the race category data ‘as is’ via one-hot encoding, as we intend to collect additional data from more patients to mitigate this problem in an upcoming study, and methods for balancing unbalanced features are not the focus of this work. It is likely that this does affect the race feature importances reported herein, as stochasticity in dividing the training, validation, and test sets may result in some races not appearing in all sets (this could be somewhat mitigated using stratified sampling; however, we did confirm that at least one case of each race was randomly selected into the test set as shown in **Table S1**). Unfortunately, the highly skewed race data was unable to provide much information to the ANN in this specific study, and as a result this category has some of the smallest importance weightings in the trained DL model (Figs. 4, 5).

We do expect that the predictive accuracy of the DL model could be improved by including additional features, although it has been rigorously demonstrated that the addition of increasing numbers of new features will first improve and then decrease model performance in the case of discrete classification [72] (recall that the DL method here results in a time series of discrete binary classifications between probability of patient survival past the time interval vs. death within the interval), and this is likely for other DL model applications as well [73]. Given the large number of possible clinical measures, the identification of the optimal set (or sets) for approaching the maximum theoretical predictive accuracy remains a substantial area of outstanding study. Fortunately, this problem is somewhat mitigated by the extensive existing body of research on clinical indicators of ICI treatment response, which should serve as guidelines for identifying features most likely to improve model performance. The accuracy of these measures and the calculated mathematical model parameters may also influence predictive accuracy, although this may be mitigated through consistent clinical measurement (this would be the case for our in-house generated data), and we are encouraged as these mathematical parameters have already been confirmed to be reliable in indicating patient response (under our assumption that response and survival are likely related).

It can be expected that the DL model would also be improved increasing the number of patients used for training. In this study, we have attempted to balance the number of features included in the analysis with the number of eligible patients. These two factors are directly at odds, with increasing feature numbers reducing the number of eligible patients. Because of our decision to include a reasonable number features that we expect to be related to ICI outcomes, we have been unable to find any fully-matched external validation sets to expand the number of eligible patients for model training in this study, and work to obtain additional in-house data is ongoing (for example, more lung patients may be obtained from NCT02444741, which ends September 2025). However, using logistic hazard-based discrete survival prediction offers some mitigation to limitations in data availability, as this approach has been reported to have superior performance than other discrete methods with small sample sizes [35]. The approach presented herein of hybridizing MBs with clinical data offers a powerful strategy to improve the accuracy of time-to-event prediction towards identifying optimal personalized treatment strategies. This platform also provides a system for rapid testing of new features (categorical, continuous, and model-derived; note that they could even be collected from multiple mechanistic models) that are suspected to be indicative of ICI response, by both weighting their contribution to increasing predictive accuracy and comparing their importance to existing measures.

## Figures and Tables

**Table 1.** Data included in the DL model. Negative values of μ and Λ occur when tumor growth rate is larger after treatment than before treatment; although the physical meaning of negative values is less intuitive, correct mathematical capture of this behavior is valuable for assessing patient prognosis.

		Parameter	Median [Range] or Count [%]

Mathematical model parameters	Continuous measures	μ (tumor kill rate; day^−1^)	0.0090 [−0.89 – 0.120]

		Λ (anti-tumor immune state; dimensionless)	0.147 [ −11.2 – 3.70]

		$\alpha_{1}$ (growth rate at 1^st^ restaging; day^−1^)	0.0085 [−0.042 – 0.064]

Clinical measures	Continuous measures	Age at time of first ICI treatment (years)	58.3 [20 – 83]

		Neutrophil count (baseline)	4.8 [0.95 – 11.91]

		Lymphocyte count (baseline)	1.16 [0.28 – 3.46]

		KPS at Date of Consult^†^	0.8 [0.6 – 1.0]

	Discrete measures	Race:	
		African American	5 [5.4%]
		Asian	3 [3.2%]
		White	76 [81.7%]
		Hispanic	9 [9.7%]

		Sex:	
		Male [code: 1]	47 [50.5%]
		Female [code: 0]	46 [49.5%]

		Histology:	
		Adenocarcinoma [code: 1]	48 [51.6%]
		Squamous Cell [code: 2]	10 [10.7%]
		Neuroendocrine [code: 3]	8 [8.6%]
		Small Cell [code: 4]*	0 [0%]
		Purely Undifferentiated [code: 5]*	0 [0%]
		Other [code: 6]	27 [29.0%]

		Ever Smoker:	
		Yes [code: 1]	52 [55.9%]
		No [code: 0]	41 [44.1%]

		Prior systemic therapy after metastasis (other than immuno):	
		Yes [code: 1]	81 [87.1%]
		No [code: 0]	12 [12.9%]

		Prior Radion:	
		Yes [code: 1]	55 [59.1%]
		No [code: 0]	38 [40.9%]

		Treatment arm:**	
		1: Concurrent Lung (50/4)	19 [20.4%]
		2: Concurrent Liver (50/4)	17 [18.2%]
		3: Sequential Lung (50/4)	20 [21.5%]
		4: Sequential Lung (50/4)	17 [18.2%]
		5: Sequential Liver/Lung (60/10)	20 [21.5%]

		Maximum adverse effect (AE) grade:	
		1	15 [16.1%]
		2	18 [19.4%]
		3	57 [61.3%]
		4	2 [2.2%]
		5	1 [1.1%]

† KPS scores were reported in 10% increments (1.0, 0.9, 0.8, 0.7, 0.9).

* After screening for patient eligibility no Small Cell or Purely undifferentiated histology patients were included in the modeling study.

** Treatment arm: patients on study NCT02239900 received stereotactic ablative radiotherapy (SABR) targeted to one or more lesions in the lung or liver, given here as (Gy/doses); all lesions treated with SABR were excluded from the total lesion burden analysis (further details may be found in [24]) to focus on ICI therapy in the mechanistic model, however treatment arms were included here under the assumption there may be some capture of abscopal effects [74].
